# Cold Shock Protein B as an Alternative to DMSO for Oocyte Vitrification

**DOI:** 10.3390/antiox15010107

**Published:** 2026-01-14

**Authors:** Xinhai Wang, Jing Guo, Kaiyan Zhang, Yi Fang, Hongyu Liu, He Ding, Yang Lyu, Xin Ma, Wenfa Lyu

**Affiliations:** 1 Key Laboratory of the Animal Production, Product Quality and Security, Ministry of Education, Jilin Agricultural University, Changchun 130117, China; 2 Jilin Provincial Key Laboratory of Beef Cattle Germplasm Resources Conservation and Utilization, Jilin Agricultural University, Changchun 130117, China; 3 Jilin Provincial International Joint Research Center of Animal Breeding and Reproduction Technology, Jilin Agricultural University, Changchun 130117, China; 4 College of Animal Science and Technology, Jilin Agricultural University, Changchun 130117, China

**Keywords:** CspB protein, DMSO, ice crystal formation suppression, oxidative stress regulation

## Abstract

Dimethyl sulfoxide (DMSO) is widely utilized in the vitrification of oocytes, but DMSO exhibits concentration-dependent toxicity, which can compromise oocyte developmental potential by disrupting key cellular processes. This study reports the first successful use of cold shock protein B (CspB protein) as a substitute for DMSO in vitrification solutions for oocyte vitrification. Combining dynamics simulations and experimental validation, we demonstrated CspB’s ability to inhibit ice crystallization and recrystallization by stabilizing its position at the ice–water interface and reducing ice formation rates. Recombinant CspB was successfully expressed and shown to bind to the oolemma. In vitrification solutions, CspB (1–2 mg/mL) effectively reduced ice crystal size and enabled a significant reduction or complete replacement of DMSO. This strategy markedly improved the post-thaw survival rates of both mouse and bovine metaphase II (MII) oocytes. Furthermore, oocytes vitrified with an optimized formulation (15% ethylene glycol + 2 mg/mL CspB) exhibited developmental competence (cleavage and blastocyst rates), oxidative stress markers (ROS, GSH), mitochondrial function (membrane potential and content), and apoptosis levels (Caspase-3/9) comparable to those treated with a standard DMSO-containing system. Transcriptomic analysis revealed that CspB’s cryoprotection involves the modulation of the mTOR signaling pathway. This role was functionally confirmed, as activation of mTOR abolished CspB’s beneficial effects, reinstating oxidative damage, mitochondrial dysfunction, and apoptosis. Thus, the CspB protein replaces DMSO with direct ice crystal formation suppression and mTOR-mediated oxidative stress regulation. This study offers a protein-based alternative to conventional permeable cryoprotectants. This approach holds promise for improving reproductive biotechnologies across species.

## 1. Introduction

Oocyte cryopreservation is pivotal for fertility preservation, yet achieving high post-thaw viability remains challenging due to inherent cellular vulnerabilities. Currently, effective strategies for cryopreservation of cells and tissues encompass slow freezing and vitrification [1]. Vitrification offers faster freezing rates and causes less damage compared to slow freezing. Dimethyl sulfoxide (DMSO), the most commonly used cryoprotectant (CPA), suppresses ice nucleation by disrupting water hydrogen bonding [2]. However, DMSO exhibits concentration-dependent toxicity, which can compromise oocyte developmental potential by disrupting key cellular processes [3]. These limitations underscore the urgent need for alternative cryoprotectants that mitigate both physical and biochemical damage during vitrification. Therefore, it is imperative to identify novel anti-freeze materials to improve traditional cryoprotectants and reduce DMSO dependence.

Over the past two decades, the discovery of antifreeze proteins has provided new ideas for establishing novel antifreeze materials. Research has demonstrated that incorporating antifreeze proteins (AFPs) into vitrification solutions significantly enhances the morphological integrity of mouse oocytes and 2-cell embryos [4]. The addition of Type III AFP to vitrification solutions improved the survival and developmental rates of mouse oocytes [5]. AFPs exhibit cryoprotective potential, but their high-dose cytotoxicity poses safety risks in embryo vitrification [6]. This limitation drives interest in cold shock proteins (CSPs), which may achieve effective cryopreservation with native biocompatibility and multi-target regulation at non-toxic concentrations. CSPs are ubiquitous in both prokaryotes and eukaryotes, specifically bind to single-stranded RNA through their conserved cold shock domain, functioning as RNA chaperones. Their primary role is to prevent and resolve secondary structures in mRNA, thereby maintaining its stability and translatability under stress conditions such as cold shock [7]. Proper storage of maternal mRNAs is essential for mammalian oocyte growth, meiotic maturation and subsequent early embryonic development [8]. In addition, researchers at Sejong University in South Korea had discovered a strain KOPRI 22228, in the Arctic, which possesses the cold shock protein B (CspB protein) with a disordered N-terminal domain, imparting remarkable antifreeze properties to the strain [9]. Given its dual functions in mRNA binding and antifreeze activity, we propose that the CspB protein has potential applications in the cryopreservation of oocytes.

Cryopreservation of biological materials, the primary concerns encompass physical disruption, chemical injury, and various cellular and molecular damages induced by freeze–thaw cycles. Physical damage often occurred, primarily due to ice crystal nucleation and recrystallization during freeze–thaw cycles mechanically disrupting oocyte membranes and subcellular structures [10]. These damages can lead to the loss of biological activity in cells or tissues, posing a major challenge for the efficient development of cryopreservation technologies [11]. Concurrently, osmotic shock and cryoprotectant toxicity elevate reactive oxygen species (ROS), triggering mitochondrial dysfunction, caspase-3 activation, and lipid peroxidation [12]. We postulated that the CspB protein may exert cryoprotective effects through direct inhibition of ice crystal growth and attenuation of oxidative.

To this end, we systematically evaluated CspB’s ice interaction properties and its functional impact on oocyte vitrification. We found that the CspB protein protects oocytes by inhibiting ice crystal formation and thereby reducing oxidative damage following cryopreservation in the absence of DMSO. Our findings establish the CspB protein as a multifunctional DMSO alternative, paving the way for developing non-toxic vitrification strategies compatible with fertility preservation.

## 2. Materials and Methods

### 2.1. Expression and Purification of CspB Protein and Fluorescent Protein GFP

All proteins in this study were recombinant. The cold shock protein CspB, based on the amino acid sequence from *Polaribacter irgensii* KOPRI 22228 discovered at Sejong University was optimized and synthesized by Sangon Biotech [8]. The optimized sequence was then cloned into the pet-28a expression vector. All proteins were expressed in *E. coli* BL21 and purified according to the method outlined by Park et al. [13]. The expression protocol for the fluorescent protein GFP followed the same procedure.

### 2.2. Ice Crystal Recrystallization

To investigate whether the CspB protein exhibits ice recrystallization inhibition activity, various concentrations of these proteins were added to physiological saline, and the sandwich recrystallization method was employed for observation. Specifically, 6 μL of each sample was prepared into a sandwich and subjected to standard crystallization procedures. The quenching temperature was set at −80 °C, with a subsequent annealing temperature of −20 °C. Observations were then conducted under a cryo-microscope [14]. Conduct statistical analysis on ice crystal diameters.

### 2.3. Molecular Simulation of CspB Protein and Water Molecular Layer

Molecular dynamics (MD) simulations of ice/water-CspB systems were conducted at temperatures of 250 K, 260 K, and 270 K using the GROMACS 2021.5 package. Homology modeling for CspB was performed using SWISS-MODEL, based on an AlphaFold-predicted template (A0A2N1FA94) [15]. The CspB protein was parameterized with the Amberff14sb force field, while water molecules were represented by the TIP4P/Ice model [16]. Notably, the melting temperature of hexagonal ice (ice Ih) in this model closely aligned with the experimental value of 273.15 K.

For the system with the primary prismatic plane exposed to liquid water, the simulation box was sized at 9.9 × 9.6 × 10.0 nm^3^. Four layers of ice Ih (consisting of 4576 water molecules) were generated using the GenIce program, with position restraints applied to the oxygen atoms throughout the MD simulation [17]. Additionally, 2552 disordered water molecules were placed approximately 0.8 nm below the ice surface and restrained to prevent icing. For blank systems, 23,458 free water molecules were added above the restrained ice Ih to observe ice formation at different temperatures. For CspB protein systems, a pre-equilibrated protein was placed 0.8 nm apart from the restrained ice, with 22,648 free water molecules added and 3 Na^+^ ions to maintain electrical neutrality [18].

Energy minimization was performed using the steepest descent algorithm, followed by annealing from 0 K to 273 K under Canonical (NVT) and Isothermal-Isobaric (NPT) ensembles. Position restraints were applied to the heavy atoms of CspB protein during this process. Subsequently, 100 ns NPT MD simulations were conducted for both blank and CspB protein systems at 250 K, 260 K, and 270 K. Pressure was maintained at 1 bar using the Berendsen barostat, and temperature was controlled using the V-rescale thermostat [19]. Bond lengths of hydrogen atoms were constrained using the LINCS algorithm, while Lennard-Jones interactions were calculated within a cutoff of 1.2 nm. Electrostatic interactions beyond this cutoff were treated using the particle-mesh Ewald (PME) method.

In this study, the CHILL+ algorithm was employed to differentiate and analyze the various ice forms observed during the simulations [20]. The simulation results were visualized using UCSF Chimera X [21].

### 2.4. Collection of Mouse Oocytes and Bovine Oocytes

Mouse oocytes were collected following intraperitoneal injections of 5 IU Pregnant Mare Serum Gonadotropin (PMSG, Ningbo Second Hormone Factory, Ningbo, China), followed by 5 IU human chorionic gonadotrophin (HCG, Ningbo Second Hormone Factory) 48 h later, with euthanasia by cervical dislocation 14–15 h post-HCG injection, all in accordance with the ethical guidelines for animal experimentation and research at Jilin Agricultural University. The oviducts were harvested to release oocyte–cumulus complexes, which were then treated with 100 IU/mL hyaluronidase to remove cumulus cells. Mature metaphase II (MII) oocytes, verified by the presence of the first polar body, were selected and transferred to M16 medium (Sigma, St. Louis, MO, USA) for incubation at 37.5 °C under 5% CO_2_ and 100% humidity for 2 h.

Bovine oocytes were sourced from local slaughterhouses and transported in saline solutions, adhering to the ethical standards of Jilin Agricultural University [22]. Oocyte–cumulus complexes (COCs) were aspirated from 3 to 8 mm follicles, with only those exhibiting three or more layers selected for culture. These COCs were washed in Medium 199 (2230823, Life, Bedford, MA, USA) supplemented with 3% fetal bovine serum (FBS, Sangon Biotech, Shanghai, China) and then transferred to a maturation medium composed of Medium 199 (11150059, Life, Bedford, MA, USA) with additions of L-glutamine (0.1 mM), estradiol (10 ng/mL), cysteamine (0.1 mM), sodium pyruvate (0.2 mM), Epidermal growth factor (EGF, 50 ng/mL), follicle-stimulating hormone (0.5 μg/mL), luteinizing hormone (0.5 μg/mL), and 10% FBS (C04002, Gemini Bio, West Sacramento, CA, USA). The COCs were matured in this medium at 38.5 °C under 5% CO_2_ and 100% humidity for 22 h.

### 2.5. Vitrification of Oocytes at MII Stage and Warming

To cryopreserve mouse oocytes using the vitrification kit (Jinan Yingli, Jinan, China), five oocytes were loaded onto each carrier straw and rapidly plunged into liquid nitrogen. A control group was established with the standard vitrification solution, while an improved vitrification solution was formulated by adding 1 mg/mL or 2 mg/mL CspB protein solution and progressively reducing the concentrations of DMSO and EG. For thawing, the mouse oocytes were processed according to the vitrification thawing kit (Jinan Yingli) instructions. Post-thaw, oocytes were transferred to M16 medium for 2 h of stabilization, and their survival rates were assessed based on morphological criteria, including membrane integrity and oocyte cytoplasm discoloration. Viable oocytes were selected for subsequent experiments.

Optimal modified vitrification conditions for mouse oocyte survival were then identified and applied to cryopreserve bovine oocytes using the same freezing and thawing procedures as for mouse oocytes. After thawing, bovine oocytes were transferred to a maturation medium for 2 h of stabilization, and their survival rates were evaluated using the same morphological criteria. Viable oocytes were advanced to subsequent experimental stages.

### 2.6. Activation of Parthenogenesis of Mouse Oocytes and In Vitro Fertilization of Bovine Oocytes

To initiate parthenogenesis in mouse oocytes, cryopreserved oocytes were transferred into a parthenogenesis activation solution consisting of Ca^2+^-free CZB supplemented with 1 mol/L SrCl_2_ and 1 mg/mL Cytochalasin B. After 6 h of culture in the activation solution, oocytes were washed three times in KSOM embryo culture medium and then incubated in KSOM at 37.5 °C, 5% CO_2_, and 100% humidity. Cleavage and blastocyst rates were recorded at 24 and 96 h post-activation, respectively.

For bovine oocytes, in vitro fertilization (IVF) and embryo culture were conducted as previously described [22]. After 22 h of maturation, MII oocytes were washed in SOF-IVF medium and then incubated with frozen-thawed semen in SOF-IVF medium. Frozen bull spermatozoa were thawed in a 37 °C water bath for 30 s and separated using a Percoll density gradient (45% and 90%) followed by centrifugation at 700× *g* for 15 min. The supernatant was discarded, and spermatozoa were resuspended in SOF-IVF medium to a concentration of 10^6^ cells/mL and added to MII oocyte droplets for incubation at 38.5 °C for 12–18 h. Fertilized oocytes were then removed from the fertilization medium and vortexed into SOFS-HEPES medium for 3 min to remove cumulus cells. Subsequently, fertilized oocytes were transferred to SOFA-aa medium and incubated under mineral oil at 38.5 °C and 5% CO_2_.

### 2.7. Intracellular ROS, GSH, and DHE Levels

To assess intracellular ROS levels, oocytes were incubated in PBS-PVP medium containing 10 µM 2′,7′-dichlorodihydrofluorescein for 20 min. Dihydroethidium (DHE) and 2′,7′-dichlorodihydrofluorescein diacetate staining were utilized for observing ROS levels. DHE, a classic fluorescent probe, is widely used for detecting ROS in tissues or cells. DHE freely enters live cells and is oxidized by reactive oxygen species to form ethidium oxide, which intercalates with chromosomal DNA to produce red fluorescence. To measure intracellular DHE levels, 15 oocytes per group were incubated in medium containing 10 µM DHE (S0063; Beyotime, Shanghai, China). For assessing glutathione (GSH) levels, 15 oocytes per group were incubated in PBS-PVP medium containing 10 µM 4-chloromethyl-6,8-difluoro-7-hydroxycoumarin (CMF2HC, Invitrogen, Waltham, MA, USA) for 30 min. A digital camera (DP72d; Olympus, Tokyo, Japan) attached to a fluorescence microscope (IX70, Olympus, Tokyo, Japan) was used for imaging. All groups of 15 oocytes followed the same procedure, including incubation, rinsing, mounting, and imaging, with consistent exposure times for all measurements. Fluorescent images of oocytes were captured and analyzed for fluorescence intensity using ImageJ software, version 7.12.

### 2.8. Intracellular JC-1 and Mito-Tracker Levels

According to previously published methods, mitochondrial membrane potential was detected using the JC-1 probe [23]. Briefly, after washing oocytes three times with SOFHEPES medium, they were placed in medium containing 0.5 µmol/L JC-1 (C2003S, Beyotime, Shanghai, China) and incubated in a 37 °C, 5% CO_2_ incubator for 30 min. Under normal mitochondrial membrane potential, JC-1 forms J-aggregates and produces red fluorescence. During apoptosis, when the mitochondrial membrane potential decreases or is lost, JC-1 exists as J-monomers and produces green fluorescence. The ratio of red to green fluorescence intensity in oocytes reflects the mitochondrial membrane potential. A total of 15 oocytes per group were analyzed. Data are presented as the mean percentage (mean ± SEM) from at least three independent experiments.

To assess the content of active mitochondria, a mitochondrial membrane potential detection kit purchased from Beyotime Biotechnology (C1071, Beyotime, Shanghai, China) was used. Oocytes were incubated in culture medium containing 2 µM Mito-Tracker Red CMXRos for 30 min. Fluorescence was observed using a digital camera connected to a fluorescence microscope. A total of 15 oocytes per group were analyzed. Image processing was performed using ImageJ software.

### 2.9. Intracellular Caspase 3 and Caspase 9 Levels

Oocytes were washed three times in PBS-PVP, fixed in 4% paraformaldehyde for one hour, and permeabilized with 0.2% Triton X-100 in PBS-PVP for 30 min. After three additional washes, the oocytes were blocked in PBS-PVP containing 3% BSA and incubated at 38.5 °C for 1.5 h. The oocytes were then incubated with caspase-3 antibody and caspase-9 antibody (1:50, Affinity, A11953, Milwaukee, WI, USA).

Following dihydrochloride (DAPI) staining, the oocytes were mounted on slides with antifade mounting medium (Beyotime, P0126, Shanghai, China) and analyzed under a Nikon Eclipse Ti-S microscope equipped with a Nikon DS-Ri1 digital camera (Nikon, Tokyo, Japan). A total of 15 oocytes per group were analyzed. Fluorescent images of the oocytes were captured and analyzed for fluorescence intensity using ImageJ software.

### 2.10. Transcriptome Sequencing

cDNA derived from oocytes was amplified employing the SMART-Seq method on the Illumina Novases™ 6000 platform, enabling the investigation of mRNA transcripts in cells at specific time points. cDNA libraries were constructed from pooled RNA of mouse oocytes in both the control and MBP groups, and sequenced on the Illumina Novases™ 6000 (Illumina, San Diego, CA, USA). RNA quality was evaluated using the miRNA Kit (Qiagen, Germantown, MD, USA). Paired-end RNA-seq was conducted, yielding a total of 1 million 2 × 150 bp reads. These raw reads, containing adapters or low-quality bases, underwent further filtering using Cut adapt 2.7 to ensure high-quality clean reads. HISAT2 2.2.0 was utilized to align reads from all samples to the mouse oocyte reference genome. String Tie, with default settings, assembled the mapped reads for each sample. Subsequently, the transcriptomes of all samples were merged to create a comprehensive transcriptome. Expression levels of all transcripts were estimated using String Tie and ballgown 3.4.0, with the abundance of mRNAs quantified by calculating the Fragments Per Kilobase of exon model per Million mapped fragments (FPKM) value. Differential gene expression analysis was performed using DESeq2 1.3.0, identifying genes with an FDR < 0.05 and an absolute fold change ≥ 2 as differentially expressed. GO and KEGG pathway enrichment analyses were performed using the DAVID 2021 database with a significance threshold of *p* < 0.05.

### 2.11. qRT-PRC Analysis

Trizol reagent (Thermo, Wilmington, DE, USA) was used to extract total cell RNA, Takara kit (Takara, Kyoto, Japan) was used for reverse transcription, and the expression of mTOR downstream genes was quantitatively detected by fuorescence. The primer sequence is shown in Table S1, and the relative expression of mRNA is calculated with 2^−ΔΔCt^.

### 2.12. Statistical Analysis

All data were analyzed using GraphPad 8.0.2 via one-way ANOVA, while immunofluorescence staining images were assessed with ImageJ. Statistical significance was set at *p* < 0.05, with *p* < 0.001 indicating high significance.

## 3. Results

### 3.1. The Function of CspB on Ice Formation

**Figure 1:**
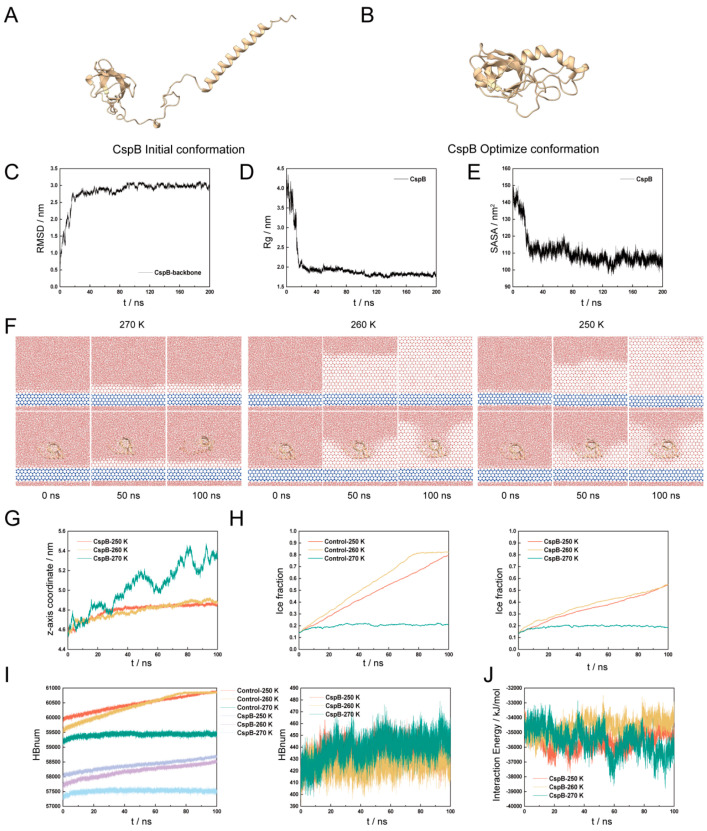
Molecular Modeling of Cold Shock Protein CspB. (**A**) Initial conformation of the CspB protein obtained through homology modeling. (**B**) Optimized conformation of the CspB protein. (**C**) RMSD of backbone atoms in the CspB protein. (**D**) Radius of gyration (Rg) of CspB protein. (**E**) Solvent-accessible surface area (SASA) of CspB protein. (**F**) Ice crystal arrangement in protein solutions at different temperatures and times. The 0 ns represents the initial point of molecular dynamics simulation of the ice water layer under different temperature conditions, therefore the three temperature conditions are the same. (**G**) Centroid coordinates of CspB along the *z*-axis. (**H**) Ice content changes under various temperature conditions. (**I**) Number of hydrogen bonds in water molecules. (**J**) Binding energy.

To provide preliminary evidence for the role of CspB in inhibiting ice crystal growth, we utilized molecular simulation techniques to focus on its interactions with water molecules and its inhibitory effects on ice crystallization. Using the PIK4 water model, we simulated ice structures at temperatures of 270 K, 260 K, and 250 K. Initially, homology modeling of the CspB protein amino acid sequence was performed based on the Alpha Fold-predicted A0A2N1FA94 template, which had 100% coverage and 74.67% sequence identity (Figure 1A). Subsequently, the simulated conformations were refined (Figure 1B), and the system’s equilibrium state was confirmed by monitoring RMSD, RG, and SASA values (Figure 1C–E).

After the system reached equilibrium, a four-layer hexagonal ice model was established as the ice nucleus, with the displacement of its O atoms restricted throughout the simulation. Additionally, the O atoms of disordered water molecules within approximately 0.8 nm below the ice seed were also immobilized to prevent ice formation on its lower surface.

No restrictions were imposed on the water molecules added above the ice seed, allowing them to move freely. Interactions between the protein and water molecules were simulated at 270 K, 260 K, and 250 K for durations of 0 ns, 50 ns, and 100 ns, respectively. The results indicated that the presence of the CspB protein in the aqueous system inhibited the formation of hexagonal ice among water molecules (Figure 1F).

To further validate our observations, we analyzed the centroid coordinates of the Z axis of the CspB protein. The results demonstrated that the protein stabilized its position within the system upon binding to ice at both 250 K and 260 K (Figure 1G). Changes in ice content serve as a key indicator of protein-mediated inhibition of ice crystal growth. We measured ice content in systems both with and without the protein at 250 K, 260 K, and 270 K. Our findings revealed that the fastest ice nucleation rate occurred at 260 K, but the addition of CspB protein reduced the ice formation rate within the system (Figure 1H).

We conducted an examination of hydrogen bonding and binding energy within the model, revealing a consistent trend between the number of intramolecular hydrogen bonds and ice content (Figure 1I). Regarding the binding energy between the CspB protein and water molecules, our analysis of the final 10 ns indicated that the system was most stable at 260 K and least stable at 270 K (Figure 1J). Preliminary molecular simulations provide evidence for the ice-inhibiting activity of the CspB protein.

### 3.2. Membrane Binding Assay of CspB Protein

**Figure 2:**
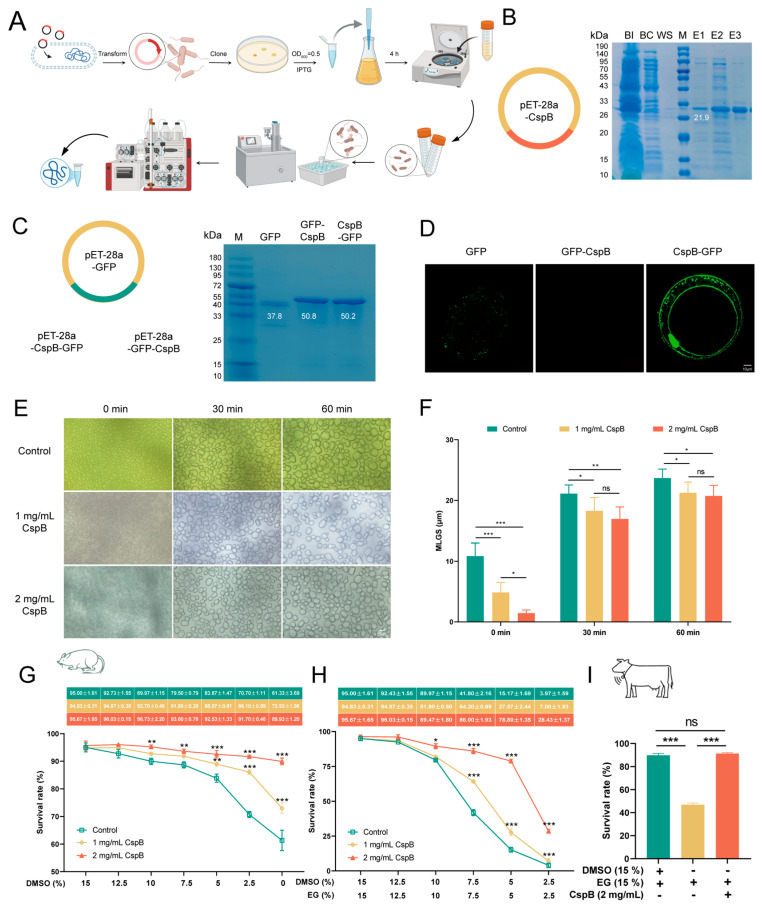
Expression and Purification of Cold Shock Protein CspB. (**A**) Protein expression process. (**B**) Recombinant plasmid of CspB, with SDS-PAGE gel electrophoresis showing the target protein expressed at 21.9 kDa. (**C**) GFP fluorescent protein and recombinant fluorescent protein plasmids, with SDS-PAGE gel electrophoresis demonstrating the expression of target proteins at 37.8, 50.8 and 50.2 kDa. (**D**) Representative images of co-culture between fluorescent protein and mouse oocytes. Fluorescent protein CspB GFP shows green fluorescence at the transparent band. (**E**) Ice recrystallization in protein solutions of different concentrations. (**F**) Ice crystal diameters at different time points and concentrations. (**G**) Survival rates of mouse MII oocytes after gradual DMSO reduction during cryopreservation. (**H**) Survival rates of mouse MII oocytes after gradual reduction of both DMSO and EG during cryopreservation. (**I**) Survival rates of bovine MII oocytes at optimal concentrations during cryopreservation. Significant differences (***, *p* < 0.001; **, *p* < 0.01; *, *p* < 0.05) are noted.

To express the CspB protein, we first reverse transcribed its amino acid sequence and ligated the resulting nucleotide sequence into the *E. coli* pET-28a expression vector (Figure 2A). The recombinant plasmid was subsequently verified through sequencing. Following successful validation, the plasmid was transformed into competent *E. coli* BL21 cells. Protein elution was performed with varying concentrations of imidazole, ultimately achieving successful elution at 300 mM imidazole, which yielded the target protein CspB at a molecular weight of 21.9 kDa (Figure 2B).

Subsequently, fluorescent GFP tags were fused to both termini of CspB. The fluorescent protein attached to the C-terminal was named CspB-GFP, and the one attached to the N-terminal was named GFP-CspB. These three sequences were individually ligated into the pET-28a vector (Figure 2C). By using repeated protein expression protocols, we successfully expressed the fluorescent proteins GFP, GFP-CspB, and CspB-GFP (Figure 2C).

To verify the binding capacity of the CspB protein with oocytes, mouse MII oocytes were exposed to M16 medium containing 2 mg/mL of fluorescent proteins for 5 min and then underwent three washes in PBS to assess fluorescence levels. The results demonstrated that the GFP fluorescent protein successfully entered the oocytes. Notably, proteins with a GFP tag attached to the N-terminus of the CspB protein failed to bind to the oocytes, while those with a GFP tag on the C-terminus of the CspB protein did (Figure 2D). These findings led us to hypothesize that the CspB protein may interact with mouse MII oocytes, thereby providing protection to the oocytes.

### 3.3. The Function of CspB Protein on Oocyte Vitrification

During the freezing and thawing process of oocytes, ice crystal formation and recrystallization were pivotal factors contributing to cellular damage.

This study investigated the impact of various concentrations of CspB protein in aqueous solution on these processes and found that CspB protein can effectively reduce the size of ice crystal recrystallization (Figure 2E). Statistical analysis of ice crystal diameters revealed that, compared to the control group without CspB protein, ice crystals in solutions containing 1 mg/mL and 2 mg/mL CspB (*p* < 0.001) were significantly smaller at the initial time (0 min) point (Figure 2F). As time progressed to 30 and 60 min, although ice crystals continued to grow, those in the two CspB protein groups remained notably smaller than those in the control group (*p* < 0.05, Figure 2F). The results indicate that the CspB protein could inhibit ice crystal recrystallization, thereby exerting a protective effect on oocytes.

In an assessment of the impact on cryopreservation survival rates of mouse MII oocytes, we introduced 1 mg/mL and 2 mg/mL of CspB protein into vitrification solutions while progressively decreasing DMSO and EG concentrations. Results showed that, with DMSO reduction alone, the addition of CspB protein significantly improved oocyte survival rates. At 0% DMSO, survival rates for the 1 mg/mL CspB group (n = 323, 72.93% ± 1.96%, *p* < 0.001) and 2 mg/mL CspB group (n = 352, 89.93% ± 1.20%, *p* < 0.001) were significantly higher than the control (n = 309, 61.33% ± 3.69%, Figure 2G). When both DMSO and EG concentrations were reduced to zero, the addition of CspB protein also could also enhance oocyte survival. Specifically, survival rates for the 1 mg/mL CspB group (n = 279, 7.00% ± 1.93%, *p* < 0.001) and 2 mg/mL CspB group (n = 288, 28.43% ± 1.37%, *p* < 0.001) were significantly elevated compared to the control (n = 297, 3.97% ± 1.59%, Figure 2H). Given the beneficial effects of the CspB protein, we selected the combination of 15% EG + 2 mg/mL CspB protein for subsequent experiments.

We also evaluated the cryopreservation survival rates of bovine MII oocytes. Compared with the vitrification group (n = 121, 85.73% ± 2.55%), the 0% DMSO group (n = 113, 46.87% ± 1.46%, *p* < 0.001) exhibited a significantly lower survival rate. However, the survival rate of the 15% EG + 2 mg/mL CspB protein group (n = 101, 87.53% ± 2.02%, *p* > 0.05) was comparable to the vitrification group (Figure 2I). These results suggested that the CspB protein can replace DMSO in vitrification solutions to enhance the cryopreservation survival rates of both mouse and bovine MII oocytes.

### 3.4. The Cryoprotective Effect of CspB Protein in Oocyte Cryopreservation

**Figure 3:**
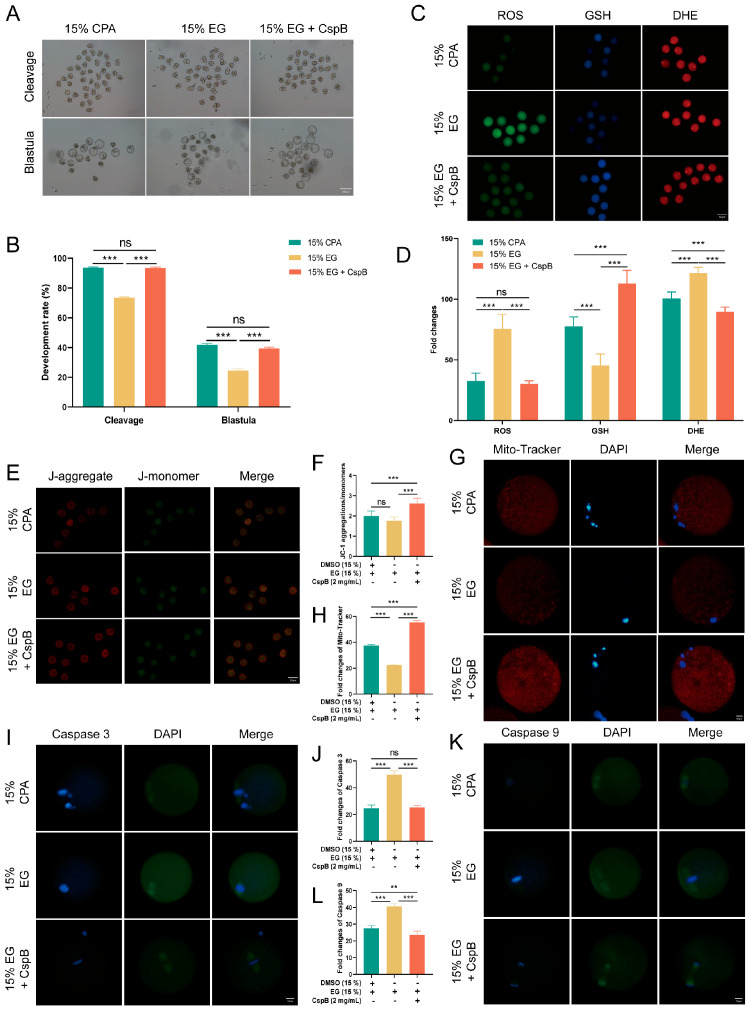
CspB Protein Enhances the Developmental Rate and Quality of Mouse MII Oocytes after Vitrification. (**A**) Representative morphologies of mouse oocytes matured after vitrification with 15% CPA, 15% EG, and 15% EG + 2 mg/mL CspB protein. (**B**) Cleavage and development rates of oocytes after parthenogenetic activation following vitrification freezing. (**C**) Representative images of ROS, GSH, and DHE in mouse oocytes after vitrification and (**D**) their fluorescence intensities. (**E**) Mitochondrial membrane potential (ΔΨm) in mouse oocytes from the three groups detected by JC-1 staining. (**F**) Ratio of red to green fluorescence intensities calculated for the three groups. (**G**) Representative images of mitochondria in mouse oocytes from the three groups and (**H**) their fluorescence intensities. (**I**) Representative images of Caspase 3 in mouse oocytes from the three groups and (**J**) their fluorescence intensities. (**K**) Representative images of Caspase 9 in mouse oocytes from the three groups and (**L**) their fluorescence intensities. Significant differences (***, *p* < 0.001; **, *p* < 0.01) are noted.

To assess the impact of CspB protein on mouse oocyte maturation, we subjected mouse MII oocytes to cryopreservation and parthenogenetic activation in 15% DMSO + 15%EG (15% CPA), 15% EG, and 15% EG + 2 mg/mL CspB groups, and evaluated their maturation rates (Figure 3A). Results revealed that, compared to the 15% CPA group (n = 159, 93.04% ± 1.70%), the cleavage rate in the 15% EG group (n = 143, 72.51% ± 0.64%, *p* < 0.001) significantly decreased, while no significant difference was observed in the 15% EG + 2 mg/mL CspB group (n = 162, 91.82% ± 0.85%, *p* > 0.05, Figure 3B). Similarly, blastocyst rates in the 15% EG group (n = 98, 24.13% ± 1.13%, *p* < 0.001) were significantly lower than those in the 15% CPA group (n = 102, 41.82% ± 1.34%), with no significant difference in the 15% EG + 2 mg/mL CspB group (n = 104, 39.37% ± 1.21%, *p* > 0.05, Figure 3B). The results indicated that 2 mg/mL of CspB protein, when used to replace DMSO in cryopreservation solutions, did not impair oocyte development potential. Furthermore, the CspB protein significantly enhances oocyte cleavage and blastocyst rates when EG is used as the cryoprotectant, indicating its potential protective role during oocyte cryopreservation.

Oxidative stress constitutes a crucial mechanism underlying oocyte damage during cryopreservation. To explore the effect of CspB protein on the cryopreservation of mouse MII oocytes, we evaluated their oxidative stress levels post-freezing using ROS, GSH, and DHE staining (Figure 3C). The results indicated that, compared to the 15% CPA group, oocytes in the 15% EG group exhibited significantly elevated ROS and DHE levels, accompanied by a notable decrease in GSH levels. However, the inclusion of 2 mg/mL CspB protein in the 15% EG group led to ROS levels that were similar to those observed in the 15% CPA group, with a significant increase in GSH levels and a significant decrease in DHE levels. The results indicate that incorporating the CspB protein into the cryopreservation medium can effectively mitigate oxidative stress in oocytes after freezing (Figure 3D).

Oxidative stress triggers the production of abundant ROS, which accumulate and impair mitochondria, leading to membrane structural damage and dysfunction. Mitochondrial membrane potential (ΔΨm) was evaluated using JC-1 (Figure 3E) staining, the results revealed that there was no significant difference in the red-to-green fluorescence ratio of oocytes between the 15% EG group and the 15% cyclopentane alcohol (CPA) group (*p* > 0.05). Nevertheless, the addition of 2 mg/mL of CspB protein to the 15% EG group (*p* < 0.001) significantly increased this ratio (Figure 3F). Furthermore, Mito-tracker (Figure 3G) staining for mitochondrial content showed a significant decrease in the 15% EG group compared to the 15% CPA group (*p* < 0.05), whereas the 15% EG + 2 mg/mL CspB group (*p* < 0.001) exhibited a significant increase in mitochondrial content (Figure 3H). Our findings suggested that the incorporation of the CspB protein into cryoprotectants can effectively reduce mitochondrial damage in oocytes after freezing.

Oxidative stress is a pivotal indicator of apoptosis. By assessing the protein expression levels of Caspase-3 and Caspase-9 (Figure 3I,K), we observed a significant elevated in both proteins in oocytes from the 15% EG group compared to the 15% CPA group (*p* < 0.001), while no notable difference was detected in the 15% EG + 2 mg/mL CspB group (*p* > 0.05) (Figure 3J,L). In summary, the incorporation of CspB protein into the cryopreservation solution effectively alleviates oxidative stress and mitochondrial damage in mouse oocytes post-freezing and inhibits the apoptotic process.

### 3.5. RNA-Seq Reveals the Mechanism of CspB-Mediated Oocyte Cryoprotection

**Figure 4:**
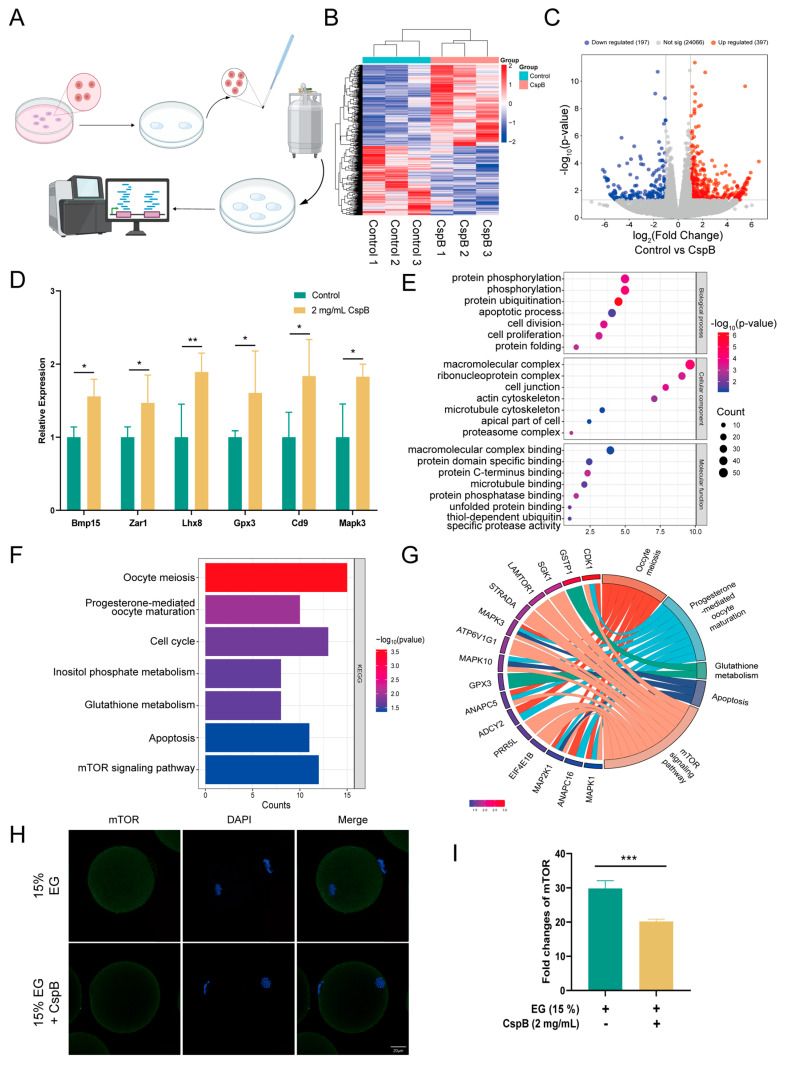
Single-Cell Transcriptome Analysis of Mouse Oocytes After Cryopreservation with 15% EG and 15% EG + 2 mg/mL CspB. (**A**) Schematic Workflow for Vitrification-Based Single-Cell Transcriptome Analysis. (**B**) Heatmap of Differentially Expressed Genes. (**C**) Volcano Plot of Differentially Expressed Genes. (**D**) Selected Upregulated Genes. (**E**) GO Enrichment Analysis. (**F**) KEGG Pathway Analysis. (**G**) Correlation Analysis of Selected Genes with KEGG Signaling Pathways. (**H**) Representative Images of mTOR in Mouse Oocytes from Both Groups and (**I**) Fluorescence Intensity Quantification. Significant differences (***, *p* < 0.001; **, *p* < 0.01; *, *p* < 0.05) are noted.

To delve deeper into the role of cold shock protein CspB in oocyte cryopreservation, we conducted RNA sequencing (RNA-seq) analysis. Specifically, we collected 30 frozen mouse MII oocytes from both the 15% EG group and the 15% EG + 2 mg/mL CspB group, with three replicates per group (Figure 4A). The results revealed significant expression differences in 594 genes. A volcano plot visually represented these differences, with 397 genes upregulated and 197 downregulated (Figure 4C). Further analysis of the differentially expressed genes showed significant upregulation (*p* < 0.05) of several key genes, including Bmp15 and Lhx8 (promoting follicular development), Zar1 and Mapk3 (promoting oocyte development), Gpx3 (protecting against oxidative stress), and Cd9 (facilitating sperm-egg fusion, Figure 4D). GO analysis revealed that the antifreeze protective effect of CspB protein is closely associated with phosphorylation, ubiquitination of proteins, and apoptotic processes in oocytes (Figure 4E). Furthermore, KEGG analysis indicated that the CspB protein is also implicated in oocyte meiosis, glutathione metabolism, and the mTOR signaling pathway (Figure 4F). Subsequently, we characterized the core genes within these signaling pathways (Figure 4G).

To further substantiate that the mTOR pathway mediates the protective effect of CspB protein on oocyte cryopreservation, we compared mTOR levels between the 15% EG group and the 15% EG + 2 mg/mL CspB group (Figure 4H). The results demonstrated that the CspB protein significantly reduced mTOR levels in cryopreserved oocytes (*p* < 0.001, Figure 4I).

### 3.6. CspB Protein Increased Vitrificated Oocyte Quality via the mTOR Pathway

**Figure 5:**
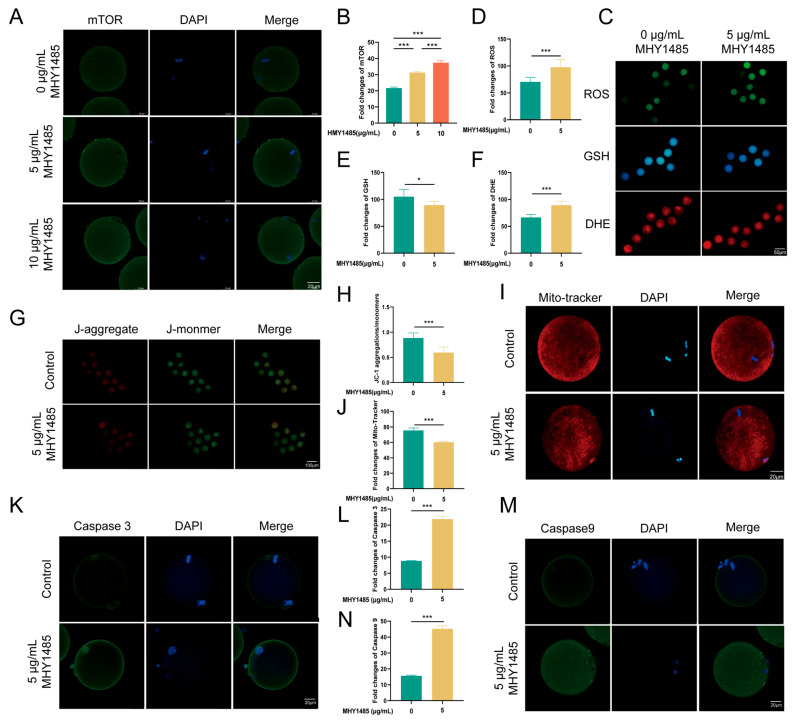
MHY1485, an mTOR activator, diminishes the quality of cryopreserved mouse oocytes treated with 15% EG + 2 mg/mL CspB. (**A**) Representative images of mTOR in mouse oocytes after the addition of various concentrations of MHY1485 and (**B**) their fluorescence intensities. (**C**) Representative images of ROS, GSH, and DHE in mouse oocytes post-MHY1485 addition. (**D**) ROS, (**E**) GSH, and (**F**) DHE fluorescence intensities in mouse oocytes with MHY1485. (**G**,**I**) Representative images of mitochondria and their fluorescence intensities (**H**,**J**) in mouse oocytes with MHY1485. (**K**) Representative images of Caspase-3 and their fluorescence intensities (**L**) in mouse oocytes with MHY1485 addition. (**M**) Representative images of Caspase-9 and their fluorescence intensities (**N**) in mouse oocytes with MHY1485. Significant differences (***, *p* < 0.001; *, *p* < 0.05) are noted.

To conduct a more in-depth exploration of the role of the mTOR pathway in the protective effect of CspB against oocyte cryopreservation injury, we conducted the following experiments. After coculturing mouse MII oocytes with varying concentrations of MHY1485 (Figure 5A), they were subjected to cryopreservation using 15% EG + 2 mg/mL CspB protein to assess oocyte quality. Results indicated that both 5 μg/mL and 10 μg/mL MHY1485 significantly upregulated mTOR expression (*p* < 0.001, Figure 5B). We selected the 5 μg/mL concentration for subsequent studies. The expression levels of mTOR downstream genes were assessed at both the mRNA and protein levels. It was found that 4EBP1 expression was down-regulated (Figure S1A–C), while SK6 expression was up-regulated (Figure S1D,E).

Upon cryopreservation of mouse MII oocytes containing this concentration of MHY1485, increased oxidative stress was observed (Figure 5C), evidenced by significant elevations in ROS and DHE levels (*p* < 0.001) and a notable decrease in GSH levels (*p* < 0.01, Figure 5D–F). Additionally, mitochondrial function was compromised, with a marked reduction in the JC-1 red-to-green fluorescence ratio and Mito-tracker labeled membrane potential (*p* < 0.001, Figure 5G–J). We also observed elevated apoptosis, with significant increases in both Caspase-3 and Caspase-9 levels (*p* < 0.001, Figure 5K–N).

### 3.7. The Function of Cspb During Bovine Oocyte Vitrification

**Figure 6:**
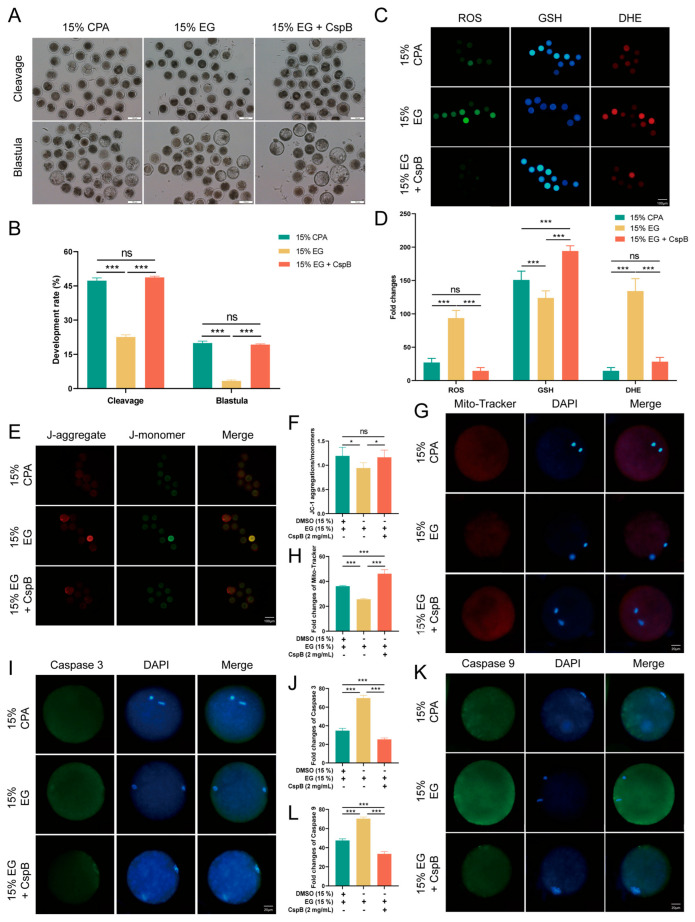
CspB Protein Enhances the Developmental Rate and Quality of Bovine MII Oocytes after Vitrification. (**A**) Representative morphologies of bovine oocytes matured after vitrification with 15% CPA, 15% EG, and 15% EG + 2 mg/mL CspB protein. (**B**) Cleavage and development rates of bovine oocytes after parthenogenetic activation following vitrification freezing. (**C**) Representative images of ROS, GSH, and DHE in bovine oocytes after vitrification and (**D**) their fluorescence intensities. (**E**) ΔΨm in oocytes from the three groups detected by JC-1 staining. (**F**) Ratio of red to green fluorescence intensities calculated for the three groups. (**G**) Representative images of mitochondria in bovine oocytes from the three groups and (**H**) their fluorescence intensities. (**I**) Representative images of Caspase-3 in bovine oocytes from the three groups and (**J**) their fluorescence intensities. (**K**) Representative images of Caspase-9 in bovine oocytes from the three groups and (**L**) their fluorescence intensities. Significant differences (***, *p* < 0.001; *, *p* < 0.05) are noted.

To investigate the effects of CspB protein on oocytes across different species, we froze bovine MII oocytes in cryopreservation media supplemented with 2 mg/mL CspB protein and evaluated their developmental potential, oxidative stress response, mitochondrial status, and apoptosis. The results indicated that compared to the 15% CPA group, the developmental rate and blastocyst rate of bovine oocytes in the 15% EG group (*p* < 0.001) significantly decreased, while those in the 15% EG + 2 mg/mL CspB protein group (*p* > 0.05) showed no significant difference (Figure 6A,B).

In terms of oxidative stress, the 15% EG group exhibited significantly elevated levels of ROS and DHE, with a decrease in GSH with the addition of the CspB protein. ROS and DHE levels were not significantly different to the control, while GSH levels increased significantly (Figure 6C,D). Regarding mitochondrial function, the 15% EG group exhibited a decreased JC-1 red/green fluorescence ratio and an increased Mito-tracker level. In contrast, the 15% EG + 2 mg/mL CspB protein group showed no significant difference in the JC-1 red/green fluorescence ratio and a marked decrease in Mito-tracker level (Figure 6E–H).

Apoptosis detection revealed significantly elevated Caspase-3 and Caspase-9 levels in the 15% EG group, compared to a notable decrease in the EG + 2 mg/mL CspB group (Figure 6I–L). In summary, the CspB protein enhances the developmental capacity of bovine oocytes during cryopreservation, alleviates oxidative stress and mitochondrial damage, and inhibits apoptosis.

## 4. Discussion

Our study pioneers the application of CspB protein as a dual-functional substitute for DMSO in oocyte vitrification, achieving enhanced oocyte cryosurvival through synergistic ice suppression and mTOR-mediated oxidative stress regulation. In this study, it was first discovered that CspB protein can replace DMSO for oocyte cryopreservation. In the absence of DMSO, the supplementation of CspB protein improved the survival and development rates of mouse and bovine oocytes post-vitrification while mitigating oxidative damage. The results observed in the CspB group were comparable to those of the control group, demonstrating its potential as an effective substitute for DMSO in cryopreservation. We systematically elucidated the antifreeze mechanisms of CspB protein, which include inhibiting ice crystal growth and alleviating oxidative damage. The results suggested that the CspB protein can serve as a novel cryoprotective additive, replacing DMSO, in oocyte cryopreservation. Specialized toxicity analysis is also necessary in applications. Lay the foundation for the development of novel cryoprotectants. These findings provide a petensial apllication for developing next-generation cryoprotectants with minimized toxicity for animal fertility preservation.

The CspB protein exhibits dual functions: mRNA binding, which stabilizes transcripts to support subsequent oocyte development, and ice crystal growth inhibition. Additionally, its N-terminal domain contains an alpha-helical structure homologous to that of AFPI, which further protects oocytes from cryo-injury. Antifreeze proteins, which inhibit ice crystal growth, are applied in the cryopreservation of various animal genetic materials. Cold shock proteins, induced under low-temperature conditions, play crucial roles in cellular survival and adaptation to cold environments, garnering our significant attention. Youn Hong Jung had identified a unique CspB protein from *P. irgensii* in Arctic, distinguished by its exceptional antifreeze activity [24]. Our objective was to express the CspB protein for utilization in the cryopreservation of oocytes. In this study, we successfully expressed the CspB protein, incorporated it into vitrification solutions, and gradually decreased the DMSO content. Notably, oocyte survival rates were significantly higher in the group with added CspB protein compared to the non-added group. Despite enhanced survival rates with different antifreeze proteins, further investigation at higher concentrations is warranted due to the challenges in protein preparation. In Yan’s study, the addition of 2.5 mg/mL of Type III antifreeze protein to the vitrification solution enhanced the cryopreservation survival rate of mouse oocytes to 92% [25]. In our research, incorporating 2 mg/mL of CspB protein into the vitrification solution elevated the survival rate to 95.67% ± 1.65. Notably, when DMSO was completely omitted from the vitrification solution, the survival rate of mouse oocytes still reached 89.93% ± 1.20. Cold shock proteins typically consist of 68 to 74 amino acids; however, the CspB protein expressed in this study comprises 150 amino acids, featuring a unique N-terminal cold shock domain [9]. This distinctive domain may play a specific role in enhancing antifreeze capability, warranting further investigation. In summary, our data indicate that the addition of CspB protein to vitrification solution enhances the survival and development rates of oocytes during cryopreservation, potentially suggesting CspB protein may serve as a substitute for DMSO in vitrification solutions. Future translational studies will involve systematic dose optimization of CspB to establish its cost-efficacy balance, ensuring maximal cryoprotection alongside commercial viability.

We uncovered a temperature-dependent ice inhibition mechanism of CspB protein, achieving superior cryoprotection at vitrification-critical temperatures (250–260 K) by dynamic ice surfaces binding and reducing hydrogen bond formation. To explore the underlying mechanism by which the CspB protein inhibits ice crystal growth, we employed molecular modeling techniques to perform homology modeling and structural optimization of the CspB protein. Simulation results indicate that CspB protein effectively inhibited suppresses ice crystal growth and reduces ice content under conditions of both 250 K and 260 K. Hydrogen bonding, a crucial factor stabilizing ice crystal structures, directly influences the stability of these structures [25]. The content of hydrogen bonding determines both the morphology and molecular arrangement of ice crystals; a higher content of hydrogen bonds results in more regular ice crystal shapes and more ordered molecular arrangements [26]. Research utilizing molecular simulation techniques has modeled the adsorption of the winter flounder antifreeze protein (wfAFP) onto ice crystals in water, revealing that the activity of the antifreeze protein is linked to hydrogen bonding and binding energy, with a higher number of hydrogen bonds conferring greater antifreeze activity [27]. The hydrogen bond content within the system with added CspB protein was significantly lower than that of the unadded group, resulting in a decreased ice content. Our study further revealed that the CspB protein inhibits ice crystal growth.

Oxidative stress is a crucial indicator of damage to oocytes during vitrification. Numerous studies have demonstrated that elevated levels of ROS can induce mitochondrial injury, potentially leading to abnormal fertilization and developmental arrest in oocytes. In our study, the removal of DMSO from vitrification solutions elevated oxidative stress levels in mouse oocytes, causing mitochondrial damage and exacerbating apoptosis. However, the addition of CspB protein to vitrification solutions reduced oxidative stress, restored mitochondrial function, and decreased apoptosis levels in oocytes. CspB protein may reduce physical damage by inhibiting ice crystal formation, which is also a crucial factor contributing to its antioxidant function. Lee’s research revealed that the incorporation of three antifreeze proteins into vitrification solutions significantly reduced ROS production in mouse oocytes, accompanied by an increase in Mito-tracker levels [5], suggesting that these proteins alleviated oxidative stress and enhanced mitochondrial function [28]. Similarly, Liang’s study found that adding antifreeze glycoprotein AFGP8 to vitrification solutions decreased ROS levels and caspase activity in bovine oocytes, indicating its ability to mitigate oxidative stress and inhibit apoptosis [29]. While our findings align with those of other researchers, the mechanism by which CspB protein enhances oocyte quality remains unclear and warrants further investigation.

The RNA-seq results specifically and significantly highlighted the mTOR signaling pathway as the top enriched pathway, generating a pivotal hypothesis that CspB coordinates the antioxidant response via mTOR regulation. The mTOR signaling pathway plays a crucial regulatory role in maintaining cellular homeostasis by modulating key processes including cell survival, oxidative stress, apoptosis, and autophagy [30]. Cai’s study revealed that β-nicotinamide mononucleotide effectively suppresses LPS-induced reactive oxygen species production in ovarian granulosa cells via modulation of the AMPK/mTOR pathway, consequently mitigating mitochondrial impairment and inhibiting apoptosis [28]. In this study, differential gene analysis of RNA-seq revealed significant enrichment of specific genes in the mTOR pathway. Subsequent validation experiments demonstrated that the CspB protein likely exerts its protective effect against oxidative damage in oocytes through the mTOR signaling pathway. RNA-seq analysis identified mTOR signaling as the central pathway modulated by CspB protein, with significant phosphorylation of its upstream effectors STRAD (1.7-fold increase, *p* < 0.01), suggesting enhanced translational control of antioxidant enzymes. mTOR, a pivotal cellular growth regulator, integrates growth factor and nutrient signals [31]. The inclusion of CspB protein in vitrification cryoprotectants can suppress the expression levels of mTOR protein. Elevated mTOR levels suppressed 4EBP1 expression and promoted S6K expression. Activation of mTOR by MHY1485 further demonstrated the suppression of 4EBP1 and promotion of S6K expression, as anticipated. This treatment also led to increased apoptosis in cryopreserved oocytes [32]. Chen’s research indicates that melatonin inhibits cell apoptosis and regulates autophagy through the AMPK/mTOR/ULK1 signaling pathway [33]. The elevation in cell apoptosis levels upon adding the mTOR activator MHY1485 aligns with the findings of this study. In this study, we investigated oocyte quality after adding mTOR activator MHY1485 to the culture medium, followed by cryopreservation with 15% EG and 2 mg/mL CspB protein. Results showed that MHY1485 exacerbated oxidative stress, induced mitochondrial damage, and promoted apoptosis in mouse oocytes. These findings suggest that CspB protein may protect oocytes from cryopreservation-induced oxidative damage via the mTOR pathway. These findings support a hierarchical mechanistic model for CspB-mediated cryoprotection. The primary action of CspB is the physical inhibition of ice formation and growth, thereby attenuating direct cryo-injury. This reduction in mechanical stress subsequently promotes intracellular stabilization, evidenced by diminished oxidative stress and organellar damage. The mTOR pathway, a central sensor of cellular homeostasis, is modulated—likely inhibited—in response to this improved physiological state [34]. This modulation in turn coordinates adaptive responses that further enhance post-thaw survival and developmental competence [34]. Thus, while mTOR activity is integral to the protective outcome, the initiating event is the ice-binding activity of CspB that mitigates primary cryodamage. This shifts the perspective in cryopreservation research from mere ice suppression towards maintaining functional molecular homeostasis at low temperatures. The hydrogen bond content within the system with added CspB protein was significantly lower than that of the unadded group, resulting in a decreased ice content. Our study further revealed that the CspB protein inhibits ice crystal growth. Thus, while mTOR activity is integral to the protective outcome, the initiating event is the ice-binding activity of CspB that mitigates primary cryodamage. This shifts the perspective in cryopreservation research from mere ice suppression towards maintaining functional molecular homeostasis at low temperatures. Future studies will be essential to determine the efficacy and mechanism of CspB in the cryopreservation of embryos.

## 5. Conclusions

In conclusion, our study demonstrates that CspB can effectively replace DMSO in oocyte vitrification, achieving comparable post-thaw survival and developmental potential. This dual function comprises two distinct mechanisms: (1) the direct inhibition of ice crystal growth to mitigate physical damage and (2) the activation of the mTOR signaling pathway alleviated oxidative stress and apoptosis. This protection is not afforded by DMSO alone.

## Data Availability

The original contributions presented in this study are included in the article and Supplementary Materials. Further inquiries can be directed to the corresponding authors.

## Supplementary Materials

The following supporting information can be downloaded at: https://www.mdpi.com/article/10.3390/antiox15010107/s1.

## Abbreviations

The following abbreviations are used in this manuscript:

DMSO	Dimethyl sulfoxide
CspB protein	Cold shock protein B
IRI	Ice recrystallization
CPA	Cryoprotectant
AFPs	Antifreeze proteins
CSPs	Cold shock proteins
ROS	Reactive oxygen species
MD	Molecular dynamics
ice Ih	Hexagonal ice
NPT	Isothermal-Isobaric
NVT	Canonical
PME	Particle-mesh Ewald
PMSG	Pregnant Mare Serum Gonadotropin
HCG	Human chorionic gonadotrophin
MII	Metaphase II
COCs	Oocyte–cumulus complexes
FBS	Fetal bovine serum
EGF	Epidermal growth factor
IVF	In vitro fertilization
DHE	Dihydroethidium
GSH	Glutathione
ΔΨm	Mitochondrial membrane potential
DAPI	Dihydrochloride
RMSD	Root mean square deviation
RNA-seq	RNA sequencing
