# Supplementary material for: Cold Shock Protein B as an Alternative to DMSO for Oocyte Vitrification
Source: Antioxidants (Basel). 2026 Jan 14;15(1):107. doi: 10.3390/antiox15010107 (PMC12837293; doi:10.3390/antiox15010107)
Supplement: Supplementary file 1 [file antioxidants-15-00107-s001.zip › antioxidants-4064827-supplementary.pdf]

Table S1. Primer sequence

Gene	Primer
4EBP1-F	CACTATACGCCACTACCCCG
4EBP1-R	CTGCTGGGAGAGTTGTGACC
SK6-F	GGCTTTTACCTAGCGCCTGA
SK6-R	CCCCCTCCTCCAGCTC

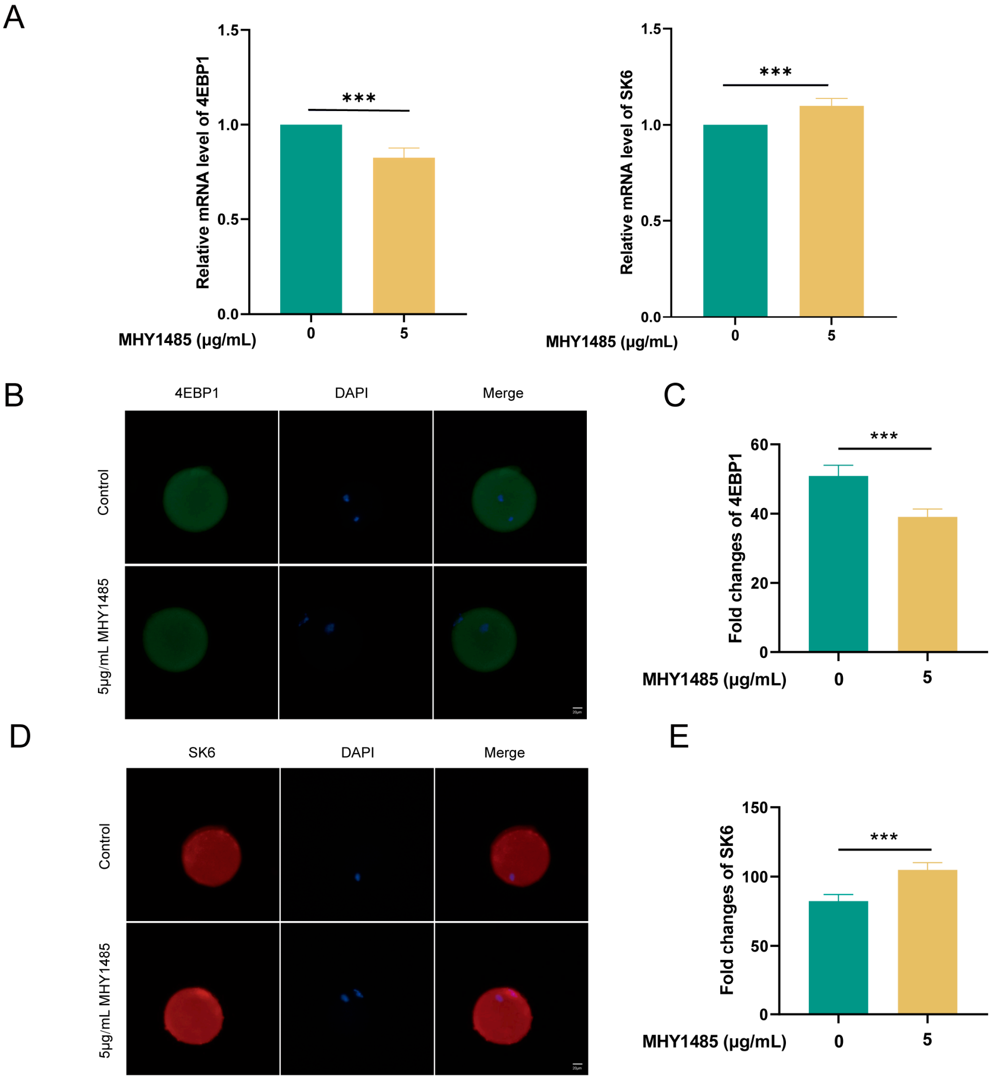

Figure S1. Effect of MHY1485 on the expression of mTOR downstream genes. (A) The mRNA expression levels of mTOR downstream genes, 4EBP1 and S6K in oocytes after CspB protein freezing using quantitative real-time PCR (qRT PCR).Representative images and quantitative analysis of 4EBP1 (B,C) and S6K (D,E) expression in mouse oocytes after cryopreservation with CspB protein.Significant differences (***, *P*<0.001; **, *P*<0.01; *, *P*<0.05) are noted.
